# Lung function and onset of cardiometabolic diseases in the longitudinal Burden of Obstructive Lung Disease study

**DOI:** 10.1136/bmjresp-2024-002442

**Published:** 2025-01-19

**Authors:** Christer Janson, James Potts, Andrei Malinovschi, Dhiraj Agarwal, Rana Ahmed, Althea Aquart-Stewart, Imed Harrabi, Meriam Denguezli, Graham Devereux, Gregory E Erhabor, Thorarinn Gislason, Rain Jogi, Sanjay K Juvekar, Ben Knox-Brown, Parvaiz Koul, Kevin Mortimer, Asaad Ahmed Nafees, Rune Nielsen, Padukudru Anand Mahesh, Stefanni Nonna M Paraguas, Anders Ørskov Rotevatn, Talant Sooronbaev, Peter G J Burney, Andre F S Amaral, BOLD Collaborative Research Group, Hasan Hafizi

**Affiliations:** 1 Department of Medical Sciences Respiratory Medicine, Uppsala Universitet, Uppsala, Sweden; 2 National Heart and Lung Institute, Imperial College London, London, UK; 3 Medical Sciences, Uppsala University, Uppsala, Sweden; 4 KEM Hospital Pune Research Centre, Pune, Maharashtra, India; 5 The Epidemiological Laboratory, Khartoum, Sudan; 6 The University of the West Indies, Kingston, Jamaica; 7 Faculté de Médecine, Sousse, Tunisia; 8 Universite de Sousse Faculte de Medecine de Sousse, Sousse, Tunisia; 9 Clinical Sciences, Liverpool School of Tropical Medicine, Liverpool, UK; 10 Department of Medicine, Obafemi Awolowo University, Ife, Nigeria; 11 Landspitali University Hospital, Reykjavik, Iceland; 12 Lung Clinic, Tartu University Hospital, Tartu, Estonia; 13 Independent Consultant, Pune, Maharashtra, India; 14 Pulmonary Medicine, SKIMS, Srinagar, India; 15 University of Cambridge, Cambridge, UK; 16 Liverpool University Hospitals NHS Foundation Trust, Liverpool, UK; 17 Community Health Sciences, Aga Khan University, Karachi, Pakistan; 18 University of Bergen, Bergen, Norway; 19 Respiratory Medicine, JSS Medical College and Hospital, Mysore, Karnataka, India; 20 Philippine College of Chest Physicians, Manila, Philippines; 21 Department of Thoracic Medicine, Haukeland University Hospital, Bergen, Norway; 22 Department of Respiratory Medicine, National Center for Cardiology and Internal Medicine, Bishkek, Kyrgyzstan; 23 Kyrgyz-Swiss High Altitude Clinic and Medical Research Center, Tuja Ashu, Kyrgyzstan

**Keywords:** Lung Physiology, Clinical Epidemiology, COPD epidemiology

## Abstract

**Introduction:**

Previous population-based studies, mainly from high-income countries, have shown that a higher forced vital capacity (FVC) is associated with a lower risk of developing cardiometabolic diseases. The aim of this study was to assess the longitudinal association between spirometry measures and the onset of cardiometabolic diseases across sites in low-income, middle-income and high-income countries.

**Methods:**

The study population comprised 5916 individuals from 15 countries participating in the Burden of Obstructive Lung Disease baseline and follow-up assessments. Postbronchodilator forced expiratory volume in 1 s (FEV1), FVC and FEV1/FVC were measured at baseline. Participants who reported having doctor-diagnosed hypertension, diabetes, heart disease and stroke at follow-up but not at baseline were considered new cases of these diseases. The association between lung function and the onset of participant-reported cardiometabolic diseases was assessed in each site using regression models, and estimates were combined using random effects meta-analysis. Models were adjusted for sex, age, smoking, body mass index and educational level.

**Results:**

Participants with greater per cent predicted FVC were less likely to have new-onset diabetes (OR per 10%=0.91, 95% CI 0.84 to 0.99), heart disease (OR per 10%=0.86, 95% CI 0.80 to 0.92) and stroke (OR per 10%=0.81, 95% CI 0.73 to 0.89) during the follow-up period (mean±SD 9.5±3.6 years). A greater percentage of FEV_1_ was associated with a lower risk of onset of heart disease and stroke. No significant association was found between FEV_1_/FVC and onset of reported cardiometabolic diseases, except for a higher risk of diabetes (OR per 10%=1.21, 95% CI 1.08 to 1.35) in participants with higher FEV_1_/FVC.

**Conclusions:**

The findings of this study suggest that a low FVC is more important than a low FEV_1_/FVC as a risk factor for developing cardiometabolic diseases. The value of including FVC in risk score models to improve their precision in predicting the onset of cardiometabolic diseases should be explored.

**WHAT IS ALREADY KNOWN ON THIS TOPIC**

- Many patients with COPD have cardiovascular disease and diabetes. Previous studies, however, suggest that cardiometabolic diseases are more closely related to low lung function than airflow obstruction. Most of these studies have been cross-sectional and are from high-income countries.

**WHAT THIS STUDY ADDS**

- This longitudinal study with almost 6000 randomly selected participants from 15 countries with different income levels showed that persons with a better lung function expressed as forced vital capacity (FVC) were less likely to develop diabetes, heart disease and stroke over 10 years.

**HOW THIS STUDY MIGHT AFFECT RESEARCH, PRACTICE OR POLICY**

- The potential value of including FVC in risk score models to improve their precision in predicting the onset of cardiometabolic diseases should be explored.

## Introduction

 Many patients with chronic obstructive pulmonary disease (COPD) have cardiovascular disease and diabetes.^1 2^ COPD is spirometrically characterised by chronic airflow obstruction. In a cross-sectional analysis of the populational-based Burden of Obstructive Lung Disease (BOLD) study, Triest *et al* found no significant association of postbronchodilator airflow obstruction with hypertension, diabetes and cardiovascular disease after adjusting for confounders.^3^ This suggests that the coexistence of chronic airflow obstruction and cardiometabolic diseases is due to shared risk factors, such as age and smoking.

Another cross-sectional BOLD analysis found an association between a low forced vital capacity (FVC) and hypertension, diabetes and cardiovascular disease.^4^ In other analyses from European subsamples of BOLD, hypertension was found to be associated with lower FVC and forced expiratory volume in 1 s (FEV_1_),^5^ and a greater pulse wave velocity was associated with both low total lung capacity and low FVC.^6^ These findings suggest that a low lung function is more important than airflow obstruction in developing cardiometabolic diseases. This is also supported by other studies showing that having a low FVC and FEV_1_ is associated with a higher risk of hypertension,^7^ diabetes,^8^,^10^ myocardial infarction and cardiovascular death.^11 12^ The biological explanation for the association is unclear, but several studies have shown an association between low FVC and FEV_1_ and systemic inflammatory markers such as fibrinogen,^11^ C reactive protein (CRP)^13 14^ and interleukin-6 (IL-6).^15 16^

The aim of this investigation was to study associations between lung function and the onset of participant-reported cardiometabolic diseases in a longitudinal setting with participants from low-income, middle-income and high-income countries. We hypothesised that the onset of hypertension, diabetes, heart disease and stroke is more closely associated with low lung function than airflow obstruction.

## Methods

### Study population

**Figure 1:**
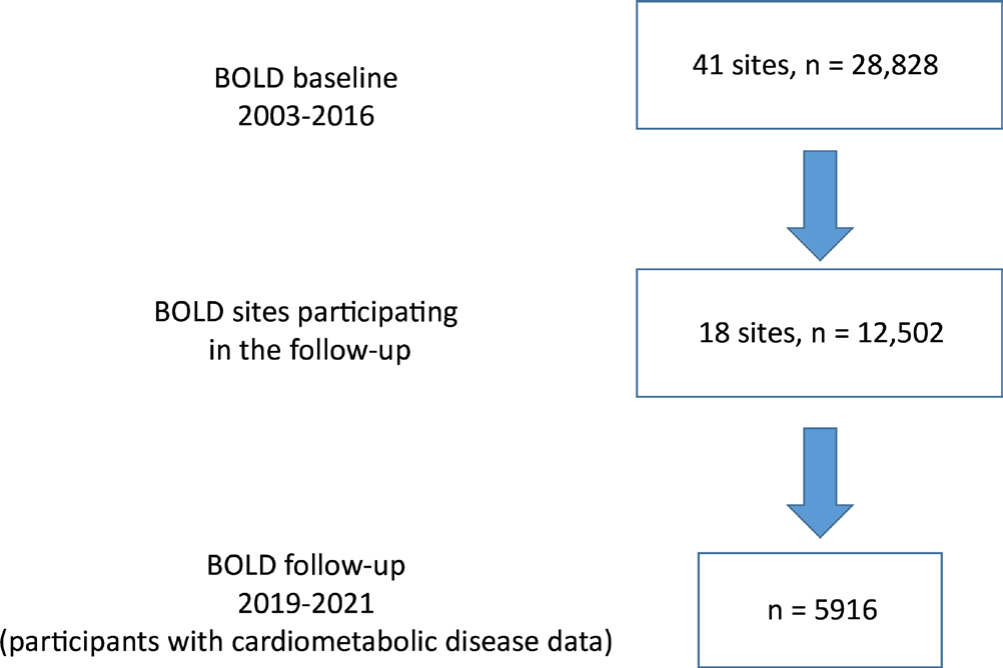
Study population of the Burden of Obstructive Lung Disease (BOLD) follow-up study.

The baseline BOLD study was conducted between 2003 and 2016 and included 41 sites across Africa, Asia, Europe, North America, the Caribbean and Oceania and collected high-quality prebronchodilator and postbronchodilator spirometry from 28 828 randomly selected participants of the age of 40 years and higher. Between 2019 and 2021, a BOLD follow-up study was conducted in 18 sites across Africa, Asia, Europe and the Caribbean. At baseline, there were 12 502 participants with high-quality spirometry at these sites. A total of 6452 participants were followed up, with 5936 completing the study core questionnaire. The current analyses included 5916 (47.3%) participants with data on cardiometabolic disease at baseline and follow-up (figure 1).^17^

### Lung function

FVC and FEV_1_ were measured at baseline before and after bronchodilation (200 µg salbutamol). The spirometry was conducted with the participant sitting upright, wearing a disposable mouthpiece and a nose clip. FEV_1_ and FVC values were obtained by spirometry using the ndd EasyOne (ndd Medizintechnik, Zurich, Switzerland). The postbronchodilator values of FVC, FEV_1_ and FEV_1_/FVC were expressed as per cent of the predicted using the reference values from the third US National Health and Nutrition Examination Survey (NHANES III) for adult European American men and women.^18^ Airflow obstruction was defined according to the lower limit of normal (LLN, <5th percentile) FEV1/FVC.^3^ Restrictive lung function was defined as having a postbronchodilator FVC below LLN.^4^

All spirometry curves were checked centrally at the BOLD Operations Centre, and usable tests had to include at least three acceptable curves, with the two best blows being within 200 mL of each other. Before the start of the surveys, study site staff underwent intensive training covering consenting, questionnaire data collection, spirometry testing and quality control, anthropometry measurements and data transfer.^17^

### Onset of cardiometabolic diseases

Participants who reported that they had been told by a doctor or healthcare provider they had hypertension, diabetes, heart disease and stroke at follow-up but not at baseline were defined as having an onset of these diseases.

The sites were classified into the level of income using the 2022–2023 World Bank Country Lending Group guidelines https://datahelpdesk.worldbank.org/knowledgebase/articles/906519-world-bank-country-and-lending-groups.

### Potential confounders

Height and weight were measured, and body mass index (BMI) was calculated as weight in kilograms divided by the square of height expressed in metres. Information on smoking history (current, ex-smoker and never-smoker), educational level and cardiometabolic diseases was obtained through a structured interview.

### Statistics

Data were analysed using Stata V.17 (Stata). Characteristics and onset of hypertension, diabetes, heart disease and stroke are presented by site and described as n (%) or mean (SD). χ^2^ test and unpaired t-test were used to compare responders and non-responders.

The association between postbronchodilator FVC, FEV_1_ and FEV_1_/FVC expressed as per cent of predicted at baseline with the onset of hypertension, diabetes, heart disease and stroke were assessed by logistic regression in each site and then combined by random effects meta-analyses. The models were adjusted for age, sex, smoking history, BMI and educational level. All regression models were adjusted for sampling weights within each site. Study sites that reported a low number of people with specific comorbidity were excluded from the meta-analysis because these sites could not be fitted into the model. Differences were considered to be significant if the p value was less than 0.05. Heterogeneity across sites was estimated using the I^2^ statistic. I^2^ values of 0%, 25%, 50% and 75% were considered no, low, moderate and high heterogeneity, respectively.

Sex-stratified analyses were performed because of previous findings of sex differences in the association between lung function and cardiometabolic diseases.^3^ Sensitivity analyses were done by excluding participants with a BMI≥30,^19^ those with any cardiometabolic disease and those with an FVC<LLN, respectively. We also used a multilevel (mixed effects) logistic regression model in our analyses. This allowed us to include participants from sites where the number of participants with comorbidities was too low to include in some of the meta-analyses.

### Patient and public involvement

Patients and/or the public were not involved in the design, or conduct, or reporting, or dissemination plans of this research.

## Results

The characteristics of the study population are presented in table 1. The mean age of the population at baseline was 54 years, and 55% of the participants were women. There was a large variation in the prevalence of current smokers and level of education across the various study sites. Participants who took part in both surveys were comparatively younger, more likely to be women, had a slightly lower BMI, were more often highly educated and had a lower prevalence of reported cardiometabolic diseases than those who only participated in the first survey. The participants also had slightly higher mean lung function values than those not participating in the follow-up study (online supplemental table S1). The mean follow-up time was 9.5 years (table 1). During this period, 1144 had an onset of hypertension, 424 had diabetes, 607 had heart disease and 154 had an onset of stroke (table 2). Of those included, 453 (7.7%) had airflow obstruction, and 2303 (38.8%) had restrictive lung function.

**Table 1:** Characteristics of the population of the BOLD follow-up study at baseline and length of follow-up (n% and mean±SD)

Country	Site	n	Age (years)	Women	BMI (kg/m^2^)	Current smokers	Follow-up (years)	Country level of income
Malawi	Chikwawa	371	53±10	188 (51)	21.8±3.8	63 (18)	6.2±4.2	Low
Sudan	Khartoum	56	52±9	24 (45)	27.1±5.5	12 (21)	8.1±2.5	Low
Benin	Seme-Kpodji	117	51±9	62 (53)	27.8±5.7	1 (1)	7.4±3.6	Low middle
Nigeria	Ife	461	55±12	322 (70)	25.8±5.7	9 (2)	8.3±3.0	Low middle
Tunisia	Sousse	269	52±8	143 (53)	29.7±5.2	62 (23)	1+0.4±1.2	Low middle
Morocco	Fes	64	53±9	28 (44)	27.7±4.9	7 (11)	10.9±1.9	Low middle
Philippines	Nampicuan-Talugtug	472	52±9	259 (55)	21.8±4.0	157 (33)	10.8±1.4	Low middle
Pakistan	Karachi	259	51±9	151 (58)	26.7±5.5	28 (11)	5.7±3.8	Low middle
India	Mysore	537	46±7	313 (58)	24.7±3.7	40 (7)	7.4±1.5	Low middle
India	Pune	694	51±9	303 (44)	22.1±3.8	51 (7)	10.9±1.5	Low middle
India	Kashmir	89	53±11	39 (43)	22.2±3.7	10 (11)	12.5±5.0	Low middle
Kyrgyzstan	Chui	469	52±9	323 (69)	28.7±5.2	88 (19)	6.2±1.8	Low middle
Kyrgyzstan	Naryn	622	53±10	392(63)	27.2±5.0	88 (14)	6.3±1.9	Low middle
Jamaica		95	56±11	53 (56)	28.8±6.9	8 (8)	5.9±1.7	High middle
Estonia	Tartu	391	58±11	199 (51)	28.2±4.9	74 (19)	10.8±1.2	High
Iceland	Reykjavik	378	52±8	171 (45)	27.9±4.6	65 (17)	15.2±1.0	High
Sweden	Uppsala	274	55±9	125 (46)	26.7±3.9	36 (13)	12.9±0.7	High
Norway	Bergen	298	54±9	138 (46)	26.3±4.0	82 (28)	15.1±0.8	High
All		5916	54±11	3233 (55)	26.0±5.5	881 (15)	9.5±3.6	

BMIbody mass indexBOLDBurden of Obstructive Lung Disease

**Table 2:** The onset of cardiometabolic disease at different sites among participants without the disease at baseline (n (%))

Country	Site	Hypertension(n=4820)	Diabetes(n=5624)	Heart disease(n=5480)	Stroke(n=5858)
Malawi	Chikwawa	20 (6)	5 (1)	11 (3)	2 (1)
Sudan	Khartoum	11 (22)	16 (29)	4 (7)	0
Benin	Seme-Kpodji	16 (19)	2 (2)	3 (3)	2 (2)
Nigeria	Ife	64 (14)	11 (2)	5 (1)	4 (1)
Tunisia	Sousse	41 (19)	43 (17)	13 (5)	9 (3)
Morocco	Fes	6 (15)	6 (11)	8 (14)	3 (5)
Philippines	Nampicuan-Talugtug	124 (33)	33 (7)	42 (10)	21 (5)
Pakistan	Karachi	57 (30)	43 (19)	13 (5)	8 (3)
India	Mysore	28 (6)	9 (2)	7 (1)	0
India	Pune	77 (12)	68 (10)	14 (2)	13 (2))
India	Kashmir	25 (40)	4 (5)	3 (3)	0
Kyrgyzstan	Chui	118 (36)	19 (4)	104 (27)	18 (4)
Kyrgyzstan	Naryn	215 (41)	43 (7)	168 (30)	19 (3)
Jamaica	Jamaica	27 (44)	27 (32)	9 (10)	7 (7)
Estonia	Tartu	72 (30)	24 (7)	53 (21)	20 (5)
Iceland	Reykjavik	99 (34)	32 (9)	72 (20)	8 (2)
Sweden	Uppsala	75 (35)	25 (9)	37 (14)	4 (1)
Norway	Bergen	69 (30)	14 (5)	41 (15)	16 (5)
**All**		1144 (24)	424 (7.5)	607 (11)	154 (3)

### Unadjusted analyses

High postbronchodilator FVC and FEV_1_ expressed as per cent of predicted at baseline were associated with a lower risk of onset of participant-reported hypertension, diabetes, heart disease and stroke. Higher FEV_1_/FVC expressed as per cent of predicted was associated with a higher risk of onset of diabetes (table 3). A moderate site heterogeneity was found for the associations with higher lung function, diabetes and stroke.

**Table 3:** Association between lung function measures as per cent of predicted and cardiometabolic diseases

	FVC	I^2^	FEV_1_	I^2^	FEV_1_/FVC	I^2^
Unadjusted						
Hypertension	**0.93 (0.88–0.98**)	0%	**0.94 (0.90–0.99**)	16%	0.95 (0.89–1.02)	24%
Diabetes	**0.85 (0.79–0.92**)	56%	**0.92 (0.87–0.99**)	49%	**1.25 (1.12–1.40**)	39%
Heart disease	**0.86 (0.80–0.92**)	0%	**0.88 (0.83–0.93**)	0%	0.94 (0.88–1.02)	13%
Stroke	**0.80 (0.73–0.88**)	55%	**0.82 (0.76–0.89**)	66%	0.98 (0.89–1.11)	54%
Adjusted						
Hypertension	0.97 (0.92–1.03)	0%	0.97 (0.92–1.02)	15%	0.94 (0.87–1.01)	24%
Diabetes	**0.91 (0.84–0.99**)	45%	0.96 (0.90–1.03)	39%	**1.21 (1.08–1.35**)	54%
Heart disease	**0.86 (0.80–0.92**)	4%	**0.88 (0.83–0.94**)	0%	0.94 (0.86–1.03)	0%
Stroke	**0.81 (0.73–0.89**)	48%	**0.83 (0.76–0.90**)	66%	1.02 (0.91–1.13)	58%

Unadjusted and adjusted odds ratioOR (95% CI CI) for 10 units change. The association was assessed in each site and then combined by random effects meta-analyses. Statistically significant estimates are marked with bold numbers. I2 values of 0%, 25%, 50%, and 75% were considered no, low, moderate, and high heterogeneity.

Adjusted for sex, age, smoke history, BMI and educational level baseline.

BMIbody mass indexFEV1, forced expiratory volume in 1 s; FVC, forced vital capacity

### Adjusted analyses

**Figure 2:**
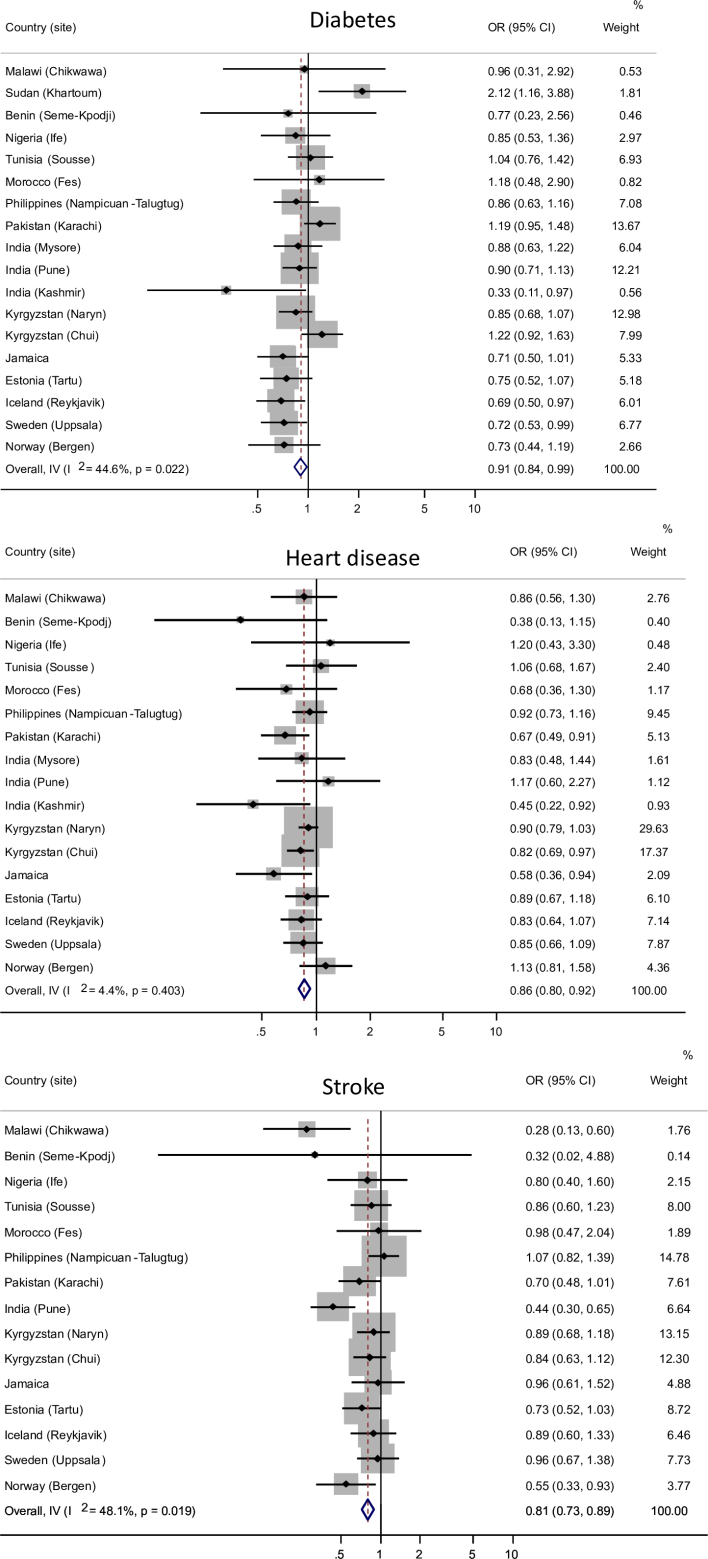
Association between forced vital capacity expressed as per cent of predicted and onset of diabetes, heart disease and stroke. Adjusted OR* (95% CI) for 10 units change. The association was assessed in each site and combined by random effects meta-analyses. I^2^ values of 0%, 25%, 50% and 75% were considered no, low, moderate and high heterogeneity. *Adjusted for age, sex, smoke history, BMI and educational level baseline. BMI, body mass index.

There was a statistically significant association between higher FVC and FEV_1_ and a lower risk of onset of heart disease and stroke after adjustment for sex, age, smoking history, BMI and educational level (table 3 and figure 2). High FVC was also associated with a lower risk of diabetes, whereas a high FEV_1_/FVCA was associated with a higher risk in the adjusted analyses. Moderate heterogeneity across sites was found for the associations of diabetes and stroke with high lung function. The association between FVC and diabetes was larger in the sites from high-income than in low-income and middle-income countries (figure 2). No income-related association was found for other associations (figure 2).

### Sensitivity analyses

In the sensitivity analyses, slight reductions were observed in the estimates when participants who were obese or had any cardiometabolic disease were excluded. Excluding participants with a BMI≥30 kg/m^2^ resulted in the association between FVC and diabetes becoming statistically non-significant (online supplemental table S2). The association between FEV_1_/FVC and diabetes remained statistically significant even after excluding participants with FVC<LLN (OR (95% CI) 1.29 (1.10 to 1.51)).

### Sex-stratified analyses

The association between a high FVC and high FEV_1_/FVC and the onset of diabetes after adjustment for sex, age, smoking history, BMI and educational level was statistically significant in men but not in women. No sex differences were found regarding the association between lung function and heart disease or stroke (online supplemental table S3). Excluding obese participants did not change these associations.

### Analyses using multilevel modelling

Analyses using multilevel logistic regression gave estimates that were very close to what was found in the models using meta-analyses (online supplemental table S4)

## Discussion

In this longitudinal study, a higher postbronchodilator FVC and FEV_1_ were associated with a decreased risk of developing participant-reported heart disease and stroke, and a higher FVC was associated with a lower risk of diabetes. The risk of developing heart disease per 10% predicted FVC decreased by 14%. For the same increase in FVC, the risk of diabetes decreased by 9%, and the risk of stroke decreased by 19%. A high FEV_1_/FVC was associated with an increased risk of developing diabetes. The association between lung function and the onset of heart disease was consistent across all geographical locations. However, there was moderate heterogeneity between sites regarding the risk of diabetes and stroke.

In the present study, participants with a high FVC had a decreased risk of having a participant-reported onset of diabetes during a mean follow-up period of approximately 10 years. This result matches Engström *et al*’s findings in a Swedish population study.^8^ They found that a low FVC was associated with insulin resistance at baseline and a higher incidence of diabetes during the follow-up.^20^ This is also in line with the results of McKeever *et al,* who reported that plasma glucose level 2 hours after oral glucose administration was inversely related to FVC and FEV_1_.^9^ An inverse association between FVC and the risk of type 2 diabetes was also found in a meta-analysis of 13 cohorts of patients with COPD.^21^ Surprisingly, we found that the association between high FVC and a lower risk of onset of diabetes was significant in men but not in women. In contrast to our results, Lee *et al* found no difference between men and women regarding the association between reduced FVC and 12-year incidence of diabetes,^22^ and Zaigham *et al* also reported a similar risk of diabetes in men and women with low lung function.^20^ We have no clear explanation for the difference between our results and those previously reported, except that the present study included participants from countries with different income levels. In contrast, previous studies have been conducted in high-income countries.

In the current study, high FVC and FEV_1_ were independently associated with a lower risk of onset of participant-reported heart disease. Previous studies have found that a low FVC is associated with increased coronary heart disease, myocardial infarction and cardiovascular death.^11 12^ The association between lung function and cardiometabolic diseases remained statistically significant in the sensitivity analysis, where we only included participants without any other cardiometabolic disease. We found no difference in the association between low lung function and heart disease between men and women. This is similar to what was reported by Lee *et al*^23^ but contrary to the findings of Schroeder *et al,* where the association between FEV_1_ and FVC and the 10-year incidence of coronary heart disease was stronger in women than men.^12^ The current study found that higher FVC and FEV_1_ were associated with a lower risk of stroke onset. This is in line with what has been reported by Li *et al* in a study using data from the UK Biobank.^10^ Previous studies have found that lower FEV_1_ and FVC were associated with signs of atherosclerosis, such as higher carotid intima–media thickness^24^ and reduced ankle-brachial pressure index.^25^

Like Lee *et al* and Cuttica *et al*,^23 26^ we found no association between cardiovascular disease and a low FEV_1_/FVC. However, we found that participants with a high FEV_1_/FVC were more likely to have an onset of diabetes. This result does not align with that of a meta-analysis of a large number of patients with COPD, where no significant association between FEV_1_/FVC and risk of type 2 diabetes was found.^21^ These results are, however, similar to the analyses of the baseline BOLD study, where airflow obstruction was inversely associated with diabetes.^3^ The association between high FEV_1_/FVC and low FVC to a higher risk of diabetes supports a recent meta-analysis that concludes that restrictive but not obstructive lung function impairment is significantly associated with type 2 diabetes.^27^ As we have not done diagnostic tests such as high-resolution CT scans, we cannot know to what extent this association with diabetes is related to interstitial lung disease or other restrictive lung disorders.

The explanation for the association between low lung function and cardiometabolic disease is not clear. It could be due to residual confounding from risk factors shared by a low lung function and cardiometabolic disease that have not been considered in the current and previous analyses. However, studies using polygenic scores^28^ and Mendelian Randomisation studies^29 30^ suggest that FVC and FEV_1_ are independent risk factors for cardiometabolic disease. Systemic inflammation could be one of the mechanisms connecting low lung function and cardiometabolic disease. In a recent study using data from two large Swedish populations, Rydell *et al* found that a low FVC and, to a lesser extent, FEV_1_ were associated with higher levels of many cardiovascular disease-linked plasma proteins. In contrast, no such association was found for FEV_1_/FVC.^16^ Likewise, Nerpin *et al*, analysing data from the NHANES population, found that a low FVC and FEV_1_ but not FEV_1_/FVC was associated with higher CRP levels.^14^ Another possibility is that the association between low lung and cardiometabolic diseases could result from suboptimal development of the lung and other organs related to environmental influences in utero and early childhood.^31^

The strengths of this study are its longitudinal design and inclusion of several geographical regions. Previous studies have mostly included participants from high-income countries, whereas the present study also includes low-income and middle-income countries. This is also the first study to examine the association between lung function and cardiometabolic diseases using postbronchodilator lung function. Another advantage is the rigorous quality control of the spirometry. The main limitation is that we use self-reported onset of cardiometabolic diseases. This is likely to be particularly problematic in low-income and middle-income countries with poorly developed health services focusing more on communicable diseases than non-communicable diseases. It is, therefore, possible that some of the associations related to diabetes and heart disease could be a consequence of undiagnosed preclinical disease. Diabetes is known to be associated with abnormal lung function, probably because of the effects of glucose on lung vasculature basement membranes.^32^ Similarly, low FVC and heart disease could reflect the effects of undiagnosed, mild, undiagnosed heart failure that is known to be associated with reduced FVC.^33^ The data also do not enable us to distinguish between different heart diseases or stroke. Another limitation is the average response rate below 50%, which we addressed using inverse probability weights. Levels of particulate matter were not included in the regression models. However, in most cases, the associations found in this study were similar across sites with high levels of particulate matter, such as Karachi, and sites with low levels of particulate matter, such as Reykjavik.^34^ Another potential limitation is that we have not adjusted for the use of corticosteroids. We know, however, from a previous publication that the proportion of participants using corticosteroids is low in this population.^35^ Therefore, it is unlikely that the use of corticosteroids has influenced our results substantially.

In the present study, high FVC was independently associated with a lower risk of participant-reported diabetes, heart disease and stroke. To our knowledge, FVC is not included in any risk score for predicting the risk of cardiometabolic events, although data also suggests that FVC predicted mortality more strongly than systolic blood pressure or BMI.^36^ Our results and several previous studies suggest that including FVC will improve the precision of risk scores used to predict the onset of diabetes and cardiovascular diseases.

## supplementary material

10.1136/bmjresp-2024-002442 online supplemental table 1

## Data Availability

Data are available on reasonable request.
