# Supplementary material for: Lung function and onset of cardiometabolic diseases in the longitudinal Burden of Obstructive Lung Disease study
Source: BMJ Open Respir Res. 2025 Jan 19;12(1):e002442. doi: 10.1136/bmjresp-2024-002442 (PMC11751964; doi:10.1136/bmjresp-2024-002442)
Supplement: online supplemental table 1 [file bmjresp-12-1-s001.docx]

**Supplementary material**

**Table S1.** Comparison of characteristics at baseline between responders and non-responders in the BOLD follow-up study (n % and mean±SD). (FEV_1_ forced expiratory volume in one second, FVC forced vital capacity)

|  | Non-responders  (n=6586) | Responders  (n=5916) | p-value |
| --- | --- | --- | --- |
| Age (years) | 56±12 | 52±10 | <0.0001 |
| Women | 3373 (51.5) | 3233 (48.9) | <0.0001 |
| BMI | 26.2±5.7 | 25.7±5.3 | <0.0001 |
| Current smoker | 946 (14.4) | 881 (14.9) | 0.39 |
| Hypertension | 1783 (27.1) | 1081 (18.3) | <0.0001 |
| Diabetes | 501 (7.6) | 278 (4.7) | <0.0001 |
| Heart disease | 530 (8.1) | 419 (7.1) | 0.04 |
| Stroke | 101 (1.5) | 43 (0.7) | <0.0001 |
| College education | 1345 (20.4) | 1773 (30.0) | <0.0001 |
| FVC % pred | 84.7±17.8 | 85.5±17.5 | 0.009 |
| FEV_1_ % pred | 84.4±19.9 | 85.8±18.5 | 0.0001 |
| FEV_1_/FVC % pred | 99.2±11.3 | 99.9±9.8 | 0.0001 |

.

**Table S2.** Analyses of the association between lung function measures as % of predicted and cardiometabolic diseases in participants with a BMI<30 and subjects without any cardiometabolic disease (hypertension, diabetes, heart disease and stroke) at baseline. Adjusted odds ratio (95% CI) for 10 units change. The association was assessed in each site and combined by random effects meta-analyses. Statistically significant estimates are marked with bold numbers.

|  |  | BMI<30 | No cardiometabolic disease |
| --- | --- | --- | --- |
| Diabetes | FVC | **0.91 (0.82-1.005)** | **0.88 (0.80-0.97)** |
|  | FEV_1_/FVC | **1.15 (1.003-1.32)** | 1.12 (0.98-1.28) |
| Heart disease | FVC | **0.86 (0.80-0.94)** | **0.89 (0.82-0.96)** |
|  | FEV_1_ | **0.88 (0.82-0.94)** | **0.90 (0.83-0.96)** |
| Stroke | FVC | **0.76 (0.67-0.87)** | **0.84 (0.73-0.96)** |
|  | FEV_1_ | **0.78 (0.70-0.86)** | **0.86 (0.76-0.96)** |

Adjusted for age, smoke history, BMI and educational level baseline

**Table S3.** Sex-stratified analyses of the association between lung function measures as % of predicted and cardiometabolic diseases. Adjusted odds ratio (95% CI) for 10 units change. The association was assessed in each site and combined by random effects meta-analyses. Statistically significant estimates are marked with bold numbers.

|  |  | Men | Women |
| --- | --- | --- | --- |
| Diabetes | FVC | **0.88 (0.78-0.99)** | 1.02 (0.92-1.12) |
|  | FEV_1_/FVC | **1.32 (1.23-1.54)** | 1.11 (0.94-1.30) |
| Heart disease | FVC | **0.84 (0.76-0.93)** | **0.86 (0.78-0.94)** |
|  | FEV_1_ | **0.86 (0.78-0.93)** | **0.89 (0.82-0.97)** |
| Stroke | FVC | **0.74 (0.64-0.85)** | **0.80 (0.71-0.90)** |
|  | FEV_1_ | **0.80 (0.72-0.90)** | **0.82 (0.74-0.90)** |

Adjusted for age, smoke history, BMI and educational level baseline

**Table S4.** Association between lung function measures as % of predicted and cardiometabolic diseases. Unadjusted and adjusted odds ratio (95% CI) for 10 units change. The estimates were calculated using multilevel (mixed effects) logistic regression. Statistically significant estimates are marked with bold numbers.

| **Adjusted** | **FVC** | **FEV_1_** | **FEV_1_/FVC** |
| --- | --- | --- | --- |
| Hypertension | 0.96 (0.82-1.01) | 0.95 (0.91-1.001) | 0.94 (0.87-1.01) |
| Diabetes | **0.89 (0.83-0.96)** | 0.95 (0.89-1.01) | **1.15 (1.02-1.30)** |
| Heart disease | **0.88 (0.83-0.95)** | **0.90 (0.84-0.95)** | 0.97 (0.88-1.07) |
| Stroke | **0.83 (0.74-0.93)** | **0.87 (0.700.96)** | 1.02 (0.86-1.21) |

Adjusted for sex, age, smoke history, BMI and educational level at baseline
